# The interference of anti-TSH autoantibody on clinical TSH detection

**DOI:** 10.3389/fendo.2024.1289923

**Published:** 2024-06-24

**Authors:** Mulin Tang, Xue Meng, Jiajia Ni, Xue Liu, Xinhui Wang, Yuchen Li, Yuwei Chai, Chunjia Kou, Li Zhang, Haiqing Zhang

**Affiliations:** ^1^ Key Laboratory of Endocrine Glucose & Lipids Metabolism and Brain Aging, Ministry of Education, Department of Endocrinology, Shandong Provincial Hospital Affiliated to Shandong First Medical University, Jinan, Shandong, China; ^2^ Department of Endocrinology and Metabolism, Zhongshan Hospital, Fudan University, Shanghai, China; ^3^ Department of Endocrinology, Shandong Provincial Hospital, Shandong University, Jinan, Shandong, China; ^4^ Department of Vascular Surgery, Shandong Provincial Hospital Affiliated to Shandong First Medical University, Shandong, Jinan, China; ^5^ Shandong Clinical Medical Center of Endocrinology and Metabolism, Jinan, China; ^6^ Institute of Endocrinology and Metabolism, Shandong Academy of Clinical Medicine, Jinan, China

**Keywords:** anti-TSH autoantibody (TSH-Ab), TSH, immunoassay interference, radioimmunoprecipitation, PEG precipitation, macro-TSH(M-TSH)

## Abstract

**Objective:**

It is well known that macro-thyroid-stimulating hormone (macro-TSH) could interfere with the detection of TSH. The anti-TSH autoantibody is an essential component of macro-TSH. However, the epidemiological characteristics and the clinical interference of the anti-TSH autoantibody are unclear.

**Methods:**

In this study, the radioimmunoprecipitation technique was used to detect the anti-TSH autoantibody. Platforms with different detection mechanisms were applied to measure the TSH in patients with the anti-TSH autoantibody. Polyethylene glycol (PEG) precipitation was used to determine the immunoassay interference.

**Results:**

The prevalence of the anti-TSH autoantibody in patients with mild subclinical hypothyroidism (SCH) and autoimmune thyroiditis, but normal thyroid function, was 4.78%. All 10 patients with anti-TSH antibodies had autoimmune diseases, with five of them having significant clinical test interference.

**Conclusion:**

The appearance of the anti-TSH antibody is not associated with thyroid autoantibodies. The presence of the anti-TSH autoantibody can interfere with the detection of TSH and can affect clinical diagnosis and treatment.

## 1 Introduction

The thyroid-stimulating hormone (TSH) is widely recognized as the most sensitive and specific test for evaluating thyroid function in the general population. Moreover, it is widely used in the screening of newborns for underactive thyroid and adults for thyroid diseases and female infertility, as well as in monitoring thyroid hormone replacement therapy (1). Close monitoring of the TSH levels is crucial in the diagnosis and treatment of thyroid disorders. However, the presence of some substances can interfere with the accurate measurement of TSH, including macro-TSH (2). The molecular weight of TSH is approximately 30 kDa, which is easily filtered by the kidneys. However, macro-TSH, with a molecular weight of approximately 150 kDa, cannot be filtered by the kidney, which ultimately accumulates in the circulatory system. Macro-TSH is biologically inactive, but remains immunoreactive. Due to the slower clearance of the high-molecular-mass TSH from the circulation, macro-TSH can result in a falsely high serum TSH concentration (3, 4). Macro-TSH is a high-molecular-weight form of TSH and has been proposed to be composed of TSH and immunoglobulins (3, 4). A study on ^125^I-TSH binding has confirmed that the immunoglobulin binding to TSH is an anti-TSH autoantibody (TSH-Ab) (5). However, the epidemiological characteristics and the clinical detection interference of TSH-Abs are not yet fully understood.

Subclinical hypothyroidism (SCH) is a common condition that can affect up to 10% of the adult population (6). Of those affected, 90% have TSH levels below 10 μIU/mL, which is considered mild SCH. The treatment of mild SCH is a topic of controversy. Currently, it is generally accepted that patients with SCH who have TSH ≥10 μIU/mL or have symptoms consistent with mild hypothyroidism should receive treatment (6). TSH is crucial for the diagnosis and treatment of SCH. In addition, the presence of TSH-Ab is often observed in patients with autoimmune thyroid disease (AITD) (7). Therefore, this study aimed to investigate the epidemiological characteristics and the clinical potential interference of TSH-Abs in patients with mild SCH and autoimmune thyroiditis but normal thyroid function.

## 2 Patients and methods

### 2.1 Patients

Serum samples were collected from patients who underwent thyroid function testing in Shandong Provincial Hospital. The control group was randomly selected from the hospital’s health checkup population, a total of 252 people (114 men and 138 women, with an average age of 44.02 ± 14.05 years). These individuals had normal thyroid function, negative thyroid autoantibodies [e.g., thyroglobulin antibody (Tg-Ab), thyroid peroxidase antibody (TPO-Ab), and TSH receptor antibody (TR-Ab)], no history of thyroid disease or family history, and no abnormality on thyroid ultrasound and physical examination of the thyroid gland. The patient group included 209 individuals with abnormal thyroid autoantibodies or SCH (with TSH levels between the upper limit of normal and 10 μIU/mL). These patients sought treatment at the endocrine clinic of Shandong Provincial Hospital (40 men and 169 women, with an average age of 46.25 ± 17.24 years). The patients were divided into three groups: group A (patients with autoimmune thyroiditis with normal thyroid function), totaling 101 cases; group B (patients with autoimmune thyroiditis with mild SCH), totaling 53 cases; and group C (patients with non-autoimmune thyroiditis with mild SCH), totaling 55 cases. The samples were stored in a −80°C refrigerator prior to analysis. This study was approved by the Biomedical Research Ethic Committee of Shandong Provincial Hospital.

### 2.2 Radioimmunoprecipitation technique

The radioimmunoprecipitation technique was utilized to detect TSH-Abs (8–10). A total of 252 control group and 209 patient group sera were sent to Beijing North Institute of Biotechnology Limited Company (Beijing, China) for testing. The experimental steps were as follows:

Of the serum, 20 μL was incubated with 100 μL (27,000 cpm/100 μL) of ^125^I-TSH (Beijing North Institute of Biotechnology Limited Company, Beijing, China) for 60 min at room temperature (23°C). A 20-μL mixture was incubated with 150 μL serum containing protein G (Merck KGaA, Darmstadt, Germany) or anti-human IgM–agarose (Sigma-Aldrich, Milan, Italy) at a concentration of 0.5% at 4°C for 24 h. The protein G and anti-IgM–agarose sera were pre-diluted 1:10 with saline containing 5% bovine serum albumin (BSA). Incubation was performed for 24 h at 4°C in a refrigerator. After detection of radioactivity, the mixture was centrifuged at 2,000 × *g* for 1 min and the supernatant aspirated. The radioactivity of the precipitation was measured.


TSH−Ab%=(precipitation 125I-TSH)/(total125I-TSH)×100%

The test was repeated and the average of the tests was taken as the sample TSH antibody titer.

### 2.3 Test for thyroid function and thyroid autoimmune antibodies

The architect immunoassay on the Architect i2000 analyzer (Abbot Diagnostics, Abbott Park, IL, USA) was used to measure the serum concentrations of TSH, FT3, FT4, and the thyroid autoantibodies (i.e., TG-Ab, TPO-Ab, and TR-Ab) in all samples. The reference intervals for FT3, FT4, and TSH are 2.43–6.01 pmol/L, 9.01–19.05 pmol/L, and 0.35–4.94 μIU/mL, respectively. The reference intervals for TG-Ab, TPO-Ab, and TR-Ab are 0–115, 0–34, and 0–1.22 IU/mL, respectively. The Elecsys® immunoassay using the Cobas e801 analyzer (Roche Diagnostics GmbH, Mannheim, Germany) was used to measure the serum concentrations of TSH in 10 patients with the anti-TSH autoantibody and again in 10 randomly selected patients without the anti-TSH autoantibody. The reference value for TSH on the Cobas e801 analyzer is 0.27–4.2 μIU/mL.

### 2.4 PEG precipitation

Polyethylene glycol (PEG) precipitation was used to determine the immunoassay interference (11). Of the serum, 200 μL was mixed with 200 μL of 25% PEG6000 (P8285; Solarbio, Beijing, China) and incubated at room temperature for 10 min. The mixture was then centrifuged at 3,500 rpm for 5 min to separate the precipitate and the supernatant was collected. The Elecsys immunoassay using the Cobas e801 analyzer (Roche Diagnostics GmbH, Mannheim, Germany) was used to measure the TSH concentrations in the supernatant. In the same way, 200 μL of serum was mixed with 200 μL of phosphate-buffered saline (PBS) as the control. It was calculated as follows:


$$\begin{array}{c} \text{Percentage of PEG-precipitable TSH}=\\ \left(\text{serum TSH }-\text{ free TSH}\right)/\left(\text{serum TSH}\right)\times 100\% \end{array}$$

### 2.5 Statistical analysis

The Kolmogorov–Smirnov test (*n* ≥ 50) or the Shapiro–Wilk test (*n*< 50) was used to test the normality of the measures. Measures obeying normal distribution were expressed as the mean ± standard deviation ( $\bar{x}$ ), while the non-normally distributed measures were expressed as the median and interquartile range (IQR). The count data were expressed as frequency (*n*) and constitutive ratio (in percent). A reference value range of < $\bar{x}$ +1.96s was used to indicate normally distributed data. The *t*-test (obeying normal distribution) or the Mann–Whitney *U* test (not obeying normal distribution) was used for comparison between groups of measured data, and the $\chi^{2}$ test or Fisher’s exact test was used for comparison between groups of count data. Excel software was utilized for the preliminary organization of data, and data description and statistical analysis were realized using SPSS 27.0 software. The statistical significance level was set at 0.05, and differences were considered statistically significant at *p*< 0.05.

## 3 Results

### 3.1 Normal value

**Figure 1:**
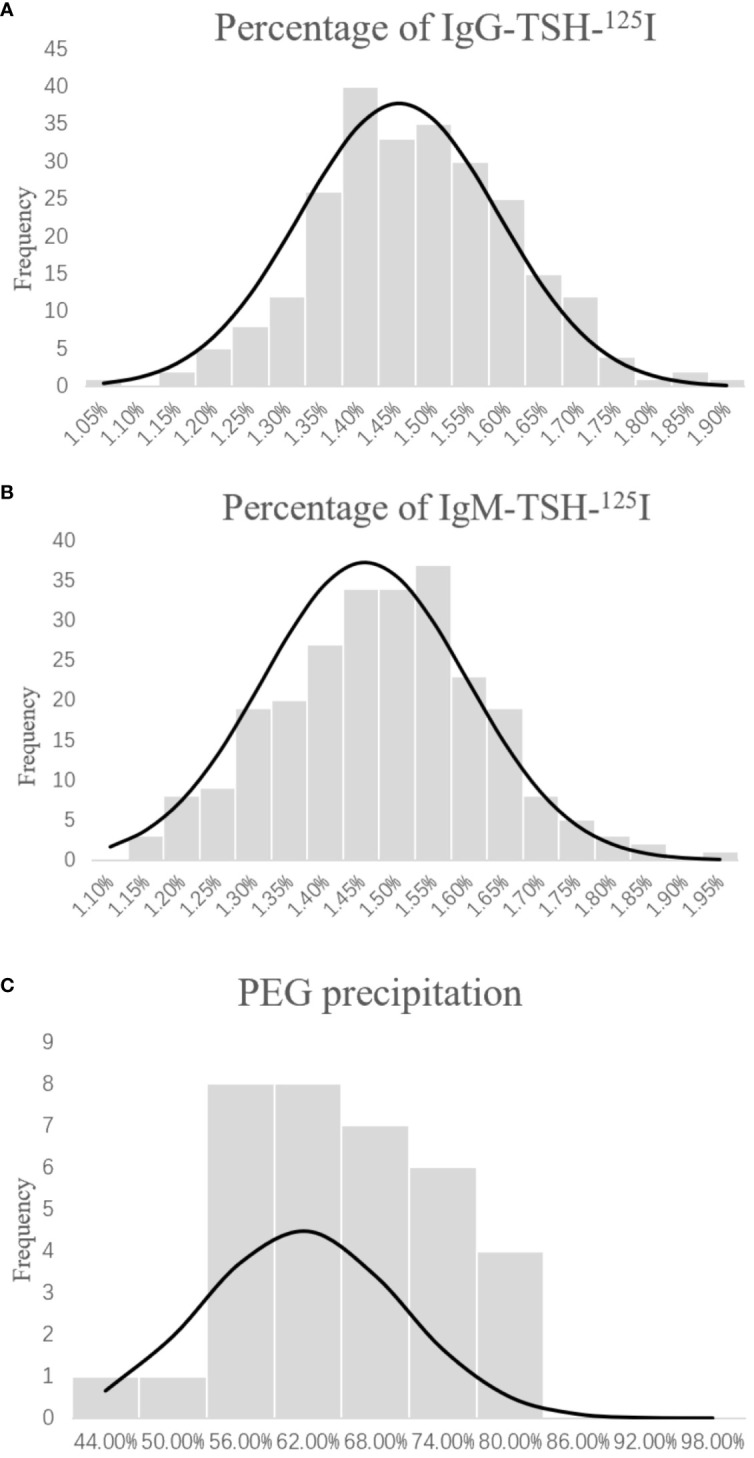
Distribution of IgG-TSH-^125^I **(A)**, IgM-TSH-^125^I **(B)**, and polyethylene glycol (PEG) sedimentation **(C)** in the healthy group. Percentage of anti-TSH autoantibody = (precipitation of ^125^I-TSH)/(total ^125^I-TSH) × 100%. Percentage of PEG-precipitable TSH = (serum TSH − free TSH)/serum TSH × 100%.

The radioimmunoprecipitation technique was used in the control group to detect the presence of TSH-Abs. The characteristics of the control group and the patient group are shown in **Table 1**. According to the Kolmogorov–Smirnov test (**Figure 1**), the IgG–TSH (1.453% ± 0.135%) and IgM–TSH (1.455% ± 0.142%) levels in the control group followed a normal distribution. The reference value range was calculated using the mean ± 1.96 SD. Antibody positivity was considered if the IgG–TSH was >1.719% or the percentage of IgM-TSH was >1.734%.

**Table 1:** Demographics and thyroid function of the healthy and patient groups.

	Control group (*n* = 252)	Patient group (*n* = 209)
Age (years)	44.02 ± 14.05	46.25 ± 17.24
Gender	M: 114; F: 138	M: 40; F: 169
FT3 (pmol/L)	4.63 ± 0.69	4.14 ± 0.55
FT4 (pmol/L)	14.67 ± 3.09	11.38 ± 1.62
TSH (µIU/ml)	2.14 ± 0.97	4.83 ± 2.72

TSH, thyroid-stimulating hormone; M, male; F, female.

### 3.2 Demographic characteristics and the prevalence of anti-TSH autoantibody

A total of 209 patient samples were used with the radioimmunoprecipitation technique to detect TSH-Abs. The total number of TSH-Ab-positive patients in the group of patients with thyroid disorders was 10, with a positivity rate of 4.78%. The TSH-Ab positivity rate in the autoimmune thyroiditis patients with normal thyroid function (group A) was 3.96% (4/101), that in autoimmune thyroiditis patients with mild SCH (group B) was 3.77% (2/53), and that in non-autoimmune thyroiditis patients with mild SCH (group C) was 7.27% (4/55). The TSH-Ab positivity rate of all patients with mild SCH was 5.55% (6/108). Of the TSH-Ab-positive patients, six were positive for IgG–TSH and four were positive for IgM–TSH. The clinical data of the 10 TSH-Ab-positive samples are presented in **Table 2**.

**Table 2:** Clinical characteristics of patients with positive anti-TSH autoantibody.

	Age (years)	Gender	Clinical diagnosis	FT3 (pmol/L)	FT4 (pmol/L)	TSH (µIU/ml)	Tg-Ab (IU/ml)	TPO-Ab (IU/ml)	TR-Ab (IU/l)	IgG–TSH	IgM–TSH
1	40	F	Hashimoto’s thyroiditis	4.24	11.05	1.496	**+**	**+**	**−**	**−**	**+**
2	13	F	Hashimoto’s thyroiditis	5.42	11.08	3.233	**+**	**+**	**−**	**−**	**+**
3	32	F	Thyroid nodule	4.07	13.25	0.857	**+**	**−**	**−**	**−**	**+**
4	51	F	Demyelinating disease	4.01	10.89	3.727	**−**	**+**	**−**	**−**	**+**
5	54	F	Hashimoto’s thyroiditis	4.63	10.17	6.053	**+**	**+**	**+**	**+**	**−**
6	24	F	Sjogren’s syndrome	5.19	9.91	6.455	**−**	**−**	**+**	**+**	**−**
7	62	F	Lung cancer with lymph node metastasis	3.60	12.65	5.978	**−**	**−**	**−**	**+**	**−**
8	58	M	Rheumatoid vasculitis	3.02	13.74	5.193	**−**	**−**	**−**	**+**	**−**
9	71	F	Lung cancer with lymph node metastasis	3.83	12.93	6.496	**−**	**−**	**−**	**+**	**−**
10	68	F	Rheumatic heart disease	3.83	12.73	8.412	**−**	**−**	**−**	**+**	**−**

TSH, thyroid-stimulating hormone; Tg-Ab, thyroglobulin antibody; TPO-Ab, thyroid peroxidase antibody; TR-Ab, TSH receptor antibody.

"+" indicates that the antibody is positive and "-" indicates that the antibody is negative.

### 3.3 Interference in TSH detection

Of the 10 TSH-Ab-positive patients, nine had higher TSH levels detected in the Roche platform compared with the Abbott platform. There were no statistical differences in gender, age, FT3, and FT4 between the 10 TSH-Ab-positive patients and the 10 randomly selected TSH-Ab-negative patients. A comparison of the basic conditions of the two groups is shown in **Table 3**. There was a statistical difference in the mean difference of the TSH levels between the two platforms (*p* = 0.008) (**Table 4**).

**Table 3:** Comparison of the baseline profiles of thyroid-stimulating hormone antibody (TSH-Ab)-positive patients and the randomly selected TSH-Ab-negative patients.

	TSH-Ab-positive patients (*n* = 10)	TSH-Ab-negative patients (*n* = 10)	*p*
Age	47.30 ± 19.39	47.40 ± 19.03	0.971
Men (*N*, %)	1 (10.00)	1 (10.00)	1.000
FT3 (pmol/L)	4.18 ± 0.72	3.93 ± 0.65	0.424
FT4 (pmol/L)	11.84 ± 1.36	11.82 ± 1.86	0.896

**Table 4:** Comparison of the thyroid-stimulating hormone (TSH) levels in TSH-Ab-positive and TSH-Ab-negative patients on different testing platforms.

Number	TSH-Ab-positive patients (*n* = 10)		TSH-Ab-negative patients (*n* = 10)	
	Abbott: TSH (µIU/mL)	Roche: TSH (µIU/mL)	Abbott: TSH (µIU/mL)	Roche: TSH (µIU/mL)
1	1.496	1.960	1.912	2.080
2	3.233	4.450	2.401	2.490
3	0.857	1.120	1.365	1.590
4	3.727	4.580	4.339	4.620
5	6.053	8.580	5.584	5.970
6	6.455	5.510	9.787	7.830
7	5.978	8.750	7.228	7.880
8	5.193	6.190	3.593	3.980
9	6.496	8.240	7.050	7.810
10	8.412	10.90	8.012	8.460
(IQR)	1.10 (0.75–2.49)		0.38 (0.21–0.67)	

IQR, interquartile range.

### 3.4 PEG precipitation

The results of the control group samples of PEG precipitation obeyed normal distribution (61.43% ± 8.88%, *p* > 0.05) (**Figure 1**). The mean + 1.96 SD (78.59%) of the PEG precipitation rate in the control group was taken as the positive cutoff point for PEG precipitation. Of the 10 TSH-Ab-positive patients, five showed positive PEG precipitation (50%). There were no statistical differences in gender, age, FT3, and FT4 between the TSH-Ab-positive patients and the 56 randomly selected TSH-Ab-negative patients (**Table 5**). The PEG precipitation positivity was 21.42% in the 56 randomly selected TSH-Ab-negative patients. There may also be other substances interfering with the detection in the TSH-Ab-negative patients, such as the heterophilic antibody; however, the PEG precipitation positivity for TSH was higher in TSH-Ab-positive patients than that in TSH-Ab-negative patients.

**Table 5:** Comparison of the clinical characteristics between thyroid-stimulating hormone antibody (TSH-Ab)-positive patients and the randomly selected TSH-Ab-negative patients.

	TSH-Ab-positive patients (*n* = 10)	TSH-Ab-negative patients (*n* = 56)	*p*
Age	47.30 ± 19.39	46.02 ± 18.49	0.795
Men (*N*, %)	1 (10.00)	12(21.42)	1.000
FT3 (pmol/L)	4.18 ± 0.72	3.93 ± 0.65	0.816
FT4 (pmol/L)	11.86 (10.71–13.01)	10.97 (9.97–11.74)	0.102

Of the five TSH-Ab-positive samples with positive PEG precipitation, three patients had IgG–TSH and two patients had IgM–TSH. The TSH concentrations of two patients (20%) were above the normal reference range on the Roche Diagnostics, but within the normal reference range on the Abbott Diagnostics. One patient (10%) showed TSH< 10 μIU/mL on the Abbott Diagnostics, but TSH > 10 μIU/mL on the Roche Diagnostics. The TSH concentrations of the remaining two patients were above the normal reference range on both the Roche and Abbott Diagnostics (**Table 6**).

**Table 6:** Thyroid-stimulating hormone (TSH) levels and polyethylene glycol (PEG) precipitation in patients with positive anti-TSH autoantibody.

Patients	Abbott: TSH (µIU/ml)	Roche: TSH (µIU/ml)	PEG supernatant TSH (µIU/ml)	PBS supernatant TSH (µIU/ml)	Sedimentation rate (%)
1	1.496	1.960	0.400	1.160	65.51
2	3.233	4.450	0.544	2.630	79.31
3	0.857	1.120	0.233	0.642	63.70
4	3.727	4.580	0.665	3.280	79.72
5	6.053	8.580	1.180	4.940	76.11
6	6.455	5.510	1.080	3.430	68.51
7	5.978	8.750	0.921	4.660	80.23
8	5.193	6.190	0.731	3.750	80.50
9	6.496	8.240	1.380	5.340	74.15
10	8.412	10.90	1.500	7.100	78.87

### 3.5 Correlation of anti-TSH autoantibodies with thyroid autoantibodies

Of the 10 TSH-Ab-positive patients in this study, the positivity rates were 40% (4/10) for Tg-Ab, 40% (4/10) for TPO-Ab, 20% (2/10) for TR-Ab, and 60% (6/10) for any thyroid autoantibodies. In 199 patients with negative TSH-Ab, the positivity rates 59.29% (118/199) for Tg-Ab, 57.78% (115/199) for TPO-Ab, 11.05% (22/199) for TR-Ab, and 74.37% (148/199) for arbitrary thyroid autoantibodies. Statistical analysis showed no statistical differences in Tg-Ab, TPO-Ab, and TR-Ab between the TSH-Ab-positive and TSH-Ab-negative patients (Tg-Ab: *p* = 0.325; TPO-Ab: *p* = 0.333; TR-Ab: *p* = 0.322; any thyroid autoantibodies: *p* = 0.295). The data are shown in **Table 7**. Therefore, it is reasonable to assume that thyroid autoantibodies have little effect on the development of TSH-Ab.

**Table 7:** Correlation of the anti-thyroid-stimulating hormone (TSH) autoantibody with thyroid autoantibodies.

Antibody	Positive^a^ (*n* = 10)	Negative^b^ (*n* = 199)	*p*
TG-Ab	40%	59.30%	0.325
TPO-Ab	40%	57.79%	0.333
TR-Ab	20%	11.06%	0.322
Any of Ab^c^	60%	74.37%	0.295

a Anti-TSH autoantibody-positive patients.

b Anti-TSH autoantibody-negative patients.

c Positive for any of the thyroid autoantibodies.

## 4 Discussion

Gel filtration chromatography (GFC) is considered the gold standard for the diagnosis of macro-TSH (2). However, it has many disadvantages: firstly, it is expensive, cumbersome, and difficult to implement in clinical practice; secondly, it can only recognize the TSH-Ab that is tightly bound to TSH and is insensitive to weak binding and free TSH-Ab (11). In addition, GFC cannot distinguish between human anti-mouse antibodies (HAMAs), anti-ruthenium antibodies, and the TSH–IgG complex (5, 11–13). Therefore, GFC does not fully meet the requirements for the clinical detection of macro-TSH and is also defective in detecting TSH-Abs.

PEG precipitation is often applied clinically to determine the presence of large molecules interfering with the test results. It is a commonly used protein precipitation method, but with low specificity. The interaction formed by PEG with proteins changes the solubility of these proteins, which can be utilized to separate proteins. Therefore, it can only determine the presence or absence of large proteins in the serum being examined, but cannot identify the presence or absence of TSH-Abs. It also cannot meet the requirements of clinical testing and is not an optimal method for the detection of TSH-Ab. On the other hand, it is not only easy to perform clinically but is also cost-effective. When it is combined with the radioimmunoprecipitation technique, it can effectively exclude detection interference. Therefore, we chose to detect TSH-Ab disturbance using both the radioimmunoprecipitation technique and PEG precipitation (8–10).

In patients with mild SCH and who have autoimmune thyroiditis but normal thyroid function, the positivity rates of TSH-Abs were higher than those of the general population. The positivity rate in women was 5.35% and that in men was 2.50%, consistent with the gender distribution of autoimmune thyroiditis (14). It has been found that most patients with macro-TSH are middle-aged or elderly, and it is thought that aging could alter the antigenicity of TSH or disrupt the body’s immune tolerance, leading to the production of TSH-Abs (5). However, in this study, the mean age of the TSH-Ab-positive patients, although higher than that of the TSH-Ab-negative patients, was not statistically different among the 10 patients who tested positive for TSH-Ab, six of whom had IgG–TSH (2.87%) and four had IgM–TSH (1.91%). Some research has provided proof that TSH-Abs can be passed from the mother to the fetus via the placenta, which is a characteristic of IgG (10, 12, 15, 16). IgG is generated by class switching from IgM. When an antigen initially appears in the body, a primary immune response occurs and IgM is produced. When the second immune response starts, IgG is generated by class switching from IgM in the presence of cytokines and T cells (17). However, previous studies have never reported the presence of IgM–TSH. IgM has a molecular weight of approximately 950 kDa, which is too large to be recognized by GFC. Previous studies only used protein G to identify TSH-Ab, which might explain why IgM–TSH has never been described before. In this research, we opted to detect TSH-Abs using the radioimmunoprecipitation technique. We firstly used anti-human IgM–agarose to precipitate IgM. Only four samples with IgM–TSH were found. IgM typically disappears after approximately 3 months, whereas IgG can persist for a long time. This might explain the number of IgM/TSH-positive samples being lower than that of IgG/TSH-positive samples. It was also observed in this research that patients with IgG–TSH were predominantly individuals with autoimmune diseases that might entail systemic multisystem damage, while patients with IgM were only those individuals with abnormal thyroid autoantibodies. However, these two antibodies showed equivalent levels of interference in TSH detection. In four patients with IgM–TSH, the PEG precipitation positivity rate was 50%, and that in six patients with IgM–TSH was also 50%.

Several epidemiological studies have shown that the prevalence of macro-TSH in SCH ranged from 0.6% to 1.62% (4, 12, 18). However, the epidemiological characteristics of TSH-Abs remain unclear. The reasons for the positive rate of TSH-Abs in this study being higher than that of macro-TSH are as follows: firstly, the laboratory technique used differed from the techniques used in previous studies. The radioimmunoprecipitation technique can recognize both the antibody binding tightly to TSH and the free TSH-Ab, while GFC only can only detect the antibody binding tightly to TSH. Secondly, GFC can only detect IgG–TSH, whereas the radioimmunoprecipitation technique can detect both IgG–TSH and IgM–TSH. Moreover, the population enrolled in this study included not only individuals with SCH but also individuals with abnormal thyroid autoantibodies, whereas the enrolled patients in previous studies only included those with SCH.

When the TSH levels were above 4 µIU/mL, the incidence of macro-TSH was 0.79% (12), with the incidence increasing to 1.67% when the TSH levels were above 10 µIU/mL (18). Previous studies have suggested that the development of macro-TSH is associated with higher TSH levels. In this study, the positivity rates of TSH-Abs were 3.96% in patients with thyroid autoantibodies and normal TSH levels and 5.55% in patients whose TSH levels were in the upper limit of normal and below 10 µIU/mL. Therefore, we speculate that, with the increase of TSH, due to more TSH exposure to the immune system, the incidence of TSH-Ab appears to increase gradually.

In the 10 TSH-Ab-positive patients in this study, six had thyroid autoantibodies, two had lymph node metastases from lung cancer undergoing immunotherapy, one had rheumatoid vasculitis, and one had rheumatic heart disease. Previous studies have suggested that Tg-Ab, TPO-Ab, and TR-Ab are more likely to be positive in patients with macro-TSH. Hence, it is necessary to pay more attention to patients who have autoimmune thyroid disorders (12). However, in our study, there was no statistical significance in the thyroid autoantibodies between the TSH-Ab-positive and TSH-Ab-negative patients. Thyroid autoantibodies have little effect on the development of TSH-Abs. The mechanism and the clinical significance of TSH-Abs are both unclear. It is possible that an epitope on the TSH molecule becomes exposed, leading to the development of TSH-Abs, although it is normally hidden. It has been reported that T-cell mitogens can cause human lymphocytes to produce immunoreactive TSH (19, 20). In this study, all of the TSH-Ab-positive patients also had other autoimmune diseases, which were not limited to AITD. Patients with an active immune response, such as those with autoimmune diseases or undergoing immunotherapy, are more likely to develop TSH-Abs.

Macro-TSH can interfere with the clinical detection of TSH, but there have been no reports of clinical detection interference by TSH-Abs. In this study, we examined the TSH of the antibody-positive patients again using the Roche platform. It was found that the mean difference in the TSH levels between the two platforms was higher in patients with TSH-Abs compared with the antibody-negative patients. TSH-Abs can bind with TSH and cause interference in detection, resulting in falsely elevated TSH concentrations, such as macro-TSH. The antibodies used in different two-site immunometric assays differ, and different antibodies have different affinity to macro-TSH, which results in the various two-site immunometric assays having different sensitivities to macro-TSH. Some platforms are more sensitive to its presence (e.g., Roche Cobas) than others (e.g., Abbott Architect) (12). However, the rate of detection interference is not clear in TSH-Ab-positive patients. To confirm TSH detection interference, 10 TSH-Ab-positive patients were investigated using PEG precipitation. Of these patients, 50% (5/10) had a positive PEG precipitation. In five TSH-Ab-positive patients with positive PEG precipitation, two had TSH levels above the normal reference range on the Roche platform, but within the normal reference range on the Abbott platform. In addition, one patient had a TSH concentration below 10 mIU/mL on the Abbott platform, but higher than 10 mIU/mL on the Roche platform. The false results of TSH can lead to misdiagnosis and unnecessary treatment and even have an impact on the clinical management of thyroid disorders.

Mild SCH is the most co mmon form of SCH. Although the TSH levels are mildly elevated, interference by TSH-Abs can still occur, resulting in potential misdiagnosis and inappropriate treatment. It is crucial to be cautious when dealing with patients who have autoimmune diseases or who are undergoing immunotherapy. Furthermore, it is important to consider the variations between the different clinical testing platforms and to take steps to eliminate the detection interference caused by TSH-Abs.

## Data availability statement

The original contributions presented in the study are included in the article/supplementary material. Further inquiries can be directed to the corresponding author.

## Ethics statement

This study was approved by the Biomedical Research Ethic Committee of Shandong Provincial Hospital. The studies were conducted in accordance with the local legislation and institutional requirements. The participants provided their written informed consent to participate in this study.

## Author contributions

MT: Writing – review & editing, Writing – original draft. XM: Writing – review & editing. JN: Writing – review & editing. XL: Writing – review & editing. XW: Writing – review & editing. YL: Writing – review & editing. YC: Writing – review & editing. CK: Writing – review & editing. LZ: Writing – review & editing. HZ: Writing – review & editing.

## References

[1] Estrada JM Soldin D Buckey TM Burman KD Soldin OP. Thyrotropin isoforms: implications for thyrotropin analysis and clinical practice. Thyroid. (2014) 24:411–23. doi: 10.1089/thy.2013.0119 PMC3949435 24073798

[2] Favresse J Burlacu MC Maiter D Gruson D. Interferences with thyroid function immunoassays: clinical implications and detection algorithm. Endocr Rev. (2018) 39:830–50. doi: 10.1210/er.2018-00119 29982406

[3] Sakai H Fukuda G Suzuki N Watanabe C Odawara M. Falsely elevated thyroid-stimulating hormone (Tsh) level due to macro-Tsh. Endocr J. (2009) 56:435–40. doi: 10.1507/endocrj.k08e-361 19336948

[4] Mills F Jeffery J Mackenzie P Cranfield A Ayling RM. An immunoglobulin G complexed form of thyroid-stimulating hormone (Macro thyroid-stimulating hormone) is a cause of elevated serum thyroid-stimulating hormone concentration. Ann Clin Biochem. (2013) 50:416–20. doi: 10.1177/0004563213476271 23828944

[5] Hattori N Ishihara T Matsuoka N Saito T Shimatsu A. Anti-thyrotropin autoantibodies in patients with macro-thyrotropin and long-term changes in macro-thyrotropin and serum thyrotropin levels. Thyroid. (2017) 27:138–46. doi: 10.1089/thy.2016.0442 27785976

[6] Biondi B Cappola AR Cooper DS. Subclinical hypothyroidism: A review. Jama. (2019) 322:153–60. doi: 10.1001/jama.2019.9052 31287527

[7] Akamizu T Mori T Imura H Noh J Hamada N Ito K. Clinical significance of anti-Tsh antibody in sera from patients with Graves' Disease and other thyroid disorders. J Endocrinol Invest. (1989) 12:483–8. doi: 10.1007/bf03350740 2571629

[8] Ni J Long Y Zhang L Yang Q Kou C Li S. High prevalence of thyroid hormone autoantibody and low rate of thyroid hormone detection interference. J Clin Lab Anal. (2022) 36:e24124. doi: 10.1002/jcla.24124 34850456 PMC8761400

[9] Benvenga S Vita R Di Bari F Lo Re C Scilipoti A Giorgianni G. Assessment of serum thyroid hormone autoantibodies in the first trimester of gestation as predictors of postpartum thyroiditis. J Clin Transl Endocrinol. (2019) 18:100201. doi: 10.1016/j.jcte.2019.100201 31428563 PMC6693681

[10] Hattori N Aisaka K Yamada A Matsuda T Shimatsu A. Prevalence and pathogenesis of macro-thyrotropin in neonates: analysis of umbilical cord blood from 939 neonates and their mothers. Thyroid. (2023) 33:45–52. doi: 10.1089/thy.2022.0457 36345221

[11] Hattori N Aisaka K Chihara K Shimatsu A. Current thyrotropin immunoassays recognize macro-thyrotropin leading to hyperthyrotropinemia in females of reproductive age. Thyroid. (2018) 28:1252–60. doi: 10.1089/thy.2017.0624 29943675

[12] Hattori N Ishihara T Shimatsu A. Variability in the detection of macro Tsh in different immunoassay systems. Eur J Endocrinol. (2016) 174:9–15. doi: 10.1530/eje-15-0883 26438715

[13] Gessl A Blueml S Bieglmayer C Marculescu R. Anti-ruthenium antibodies mimic macro-Tsh in electrochemiluminescent immunoassay. Clin Chem Lab Med. (2014) 52:1589–94. doi: 10.1515/cclm-2014-0067 24829195

[14] Antonelli A Ferrari SM Corrado A Di Domenicantonio A Fallahi P. Autoimmune thyroid disorders. Autoimmun Rev. (2015) 14:174–80. doi: 10.1016/j.autrev.2014.10.016 25461470

[15] Donadio-Andrei S Hubert N Raverot V Plantin-Carrenard E Kuczewski E Charrié A. A challenging case: highly variable Tsh in a mother and her two children. Clin Chem Lab Med. (2019) 57:e114–e7. doi: 10.1515/cclm-2018-0871 30375344

[16] Rix M Laurberg P Porzig C Kristensen SR. Elevated thyroid-stimulating hormone level in a euthyroid neonate caused by macro thyrotropin-Igg complex. Acta Paediatr. (2011) 100:e135–7. doi: 10.1111/j.1651-2227.2011.02212.x 21352360

[17] Chaplin DD. Overview of the immune response. J Allergy Clin Immunol. (2010) 125:S3–23. doi: 10.1016/j.jaci.2009.12.980 20176265 PMC2923430

[18] Hattori N Ishihara T Yamagami K Shimatsu A. Macro Tsh in patients with subclinical hypothyroidism. Clin Endocrinol (Oxf). (2015) 83:923–30. doi: 10.1111/cen.12643 25388002

[19] Smith EM Phan M Kruger TE Coppenhaver DH Blalock JE. Human lymphocyte production of immunoreactive thyrotropin. Proc Natl Acad Sci U.S.A. (1983) 80:6010–3. doi: 10.1073/pnas.80.19.6010 PMC534349 6351072

[20] Noh J Hamada N Saito H Oyanagi H Ishikawa N Momotani N. Evidence against the importance in the disease process of antibodies to bovine thyroid-stimulating hormone found in some patients with Graves' Disease. J Clin Endocrinol Metab. (1989) 68:107–13. doi: 10.1210/jcem-68-1-107 2909548

